# ‘[M]ercy is justice…and should not be denied’: Lord Dawson, the British medico-legal community, and the Infanticide Act, 1938

**DOI:** 10.1017/mdh.2025.3

**Published:** 2025-04

**Authors:** Kelly-Ann Couzens

**Affiliations:** 1 Faculty of Arts, University of Warwick, Coventry, UK, CV4 7AL; 2 Honorary Research Fellow, Department of History, University of Western Australia

**Keywords:** Lord Dawson, *R v. Hale*, Infanticide Act, 1938 inter-war Britain, medico-legal, motherhood

## Abstract

In December 1937, influential physician and politician Lord Dawson of Penn introduced an Infanticide Bill into the House of Lords. Seven months later, following minor amendments, Dawson’s Bill passed into law as the Infanticide Act, 1938. This legislation significantly altered the earlier provisions of the Infanticide Act, 1922, which introduced the offence of infanticide into English and Welsh courtrooms for the first time. Under Dawson’s reforms, a woman could be found guilty of infanticide rather than capital murder if the killing of her child, aged no more than one year old, could be attributed to a disturbance in the balance of the mother’s mind following childbirth or from lactation. Although the language and implications of the 1938 Act have ignited significant debate within legal scholarship, the creation of Dawson’s Bill and the leading role medical practitioners played in its enactment have received limited attention from historians. This article helps to address this gap by analyzing the critical response of the inter-war British medical profession to the question of infanticide reform against a backdrop of growing psychiatric ambivalence about a causal link between insanity and female reproductive states. Crucially, this paper contends that ancillary concerns over citizenship, motherhood, and the health of the nation informed Dawson’s motivations and justification for infanticide reform during the 1930s. It also seeks to foreground the physician’s distinct contribution to the birth of the 1938 Act by underscoring his efforts in devising and promoting the Bill within Parliament and among inter-war medical and legal communities.

In the early hours of 31 May 1936, Hertfordshire police received a telephone message to attend a violent incident at a farm called ‘Akaroa’, near the village of Welwyn. On arrival at the scene, the local constable was met by obstetric-surgeon Charles Medlock, who had been called in by nursing attendants to provide emergency care for a male infant and his mother, 26-year-old housewife Brenda Hale. Hale had slit the throat of her 3-week-old son and made an attempt on her own life shortly after 6 am. Dr Medlock led the officer to the farmhouse bathroom, where, wrapped in a towel and placed in the bathtub, the now lifeless infant lay.^1^ Medlock noted the extreme distress of the young mother following the attack, recalling: ‘Mrs Hale asked me in a whisper if I could possibly end her life and inquired if the baby was dead’.^2^ The police recovered a bloodied razor and two notes in Hale’s hand from the crime scene. On one of these, she had written: ‘I only want to die. Can’t I be quietly put away?’^3^

A little over seven weeks after the incident, Brenda Hale was brought to trial at the Old Bailey on the charges of attempted suicide and for the murder of her infant son.^4^ Although Hale’s note expressed a desire for the incident to be handled discretely, the proceedings against her garnered extensive press attention. In particular, the expert opinion given by defence medical witness Lord Dawson of Penn^5^ as to the nature of the offence and Hale’s mental state at the time of the crimes was reported at length in the British tabloids and medical and legal journals.^6^ Time would reveal Dawson’s involvement in the case to be auspicious. His testimony contributed favourably to the acquittal of the young mother for murder on the grounds of insanity. The trial also marked the beginning of Dawson’s successful personal campaign to secure reform of the law on infanticide in England and Wales. Indeed, eighteen months after the Hale trial, Dawson introduced his own Infanticide Bill into the House of Lords. In June 1938, the Bill was passed into law with only limited amendments, as the Infanticide Act 1938.^7^

The Infanticide Act 1938 was the second piece of inter-war legislation to establish the crime of infanticide in England and Wales.^8^ As such, it significantly expanded upon the Infanticide Act 1922, which first introduced the offence into law. Under the earlier law, a mother could be found guilty of infanticide if she had killed her ‘newly-born child’ while the ‘balance of her mind’ was ‘disturbed’ following its birth.^9^ Such women were to be dealt with as though they had been found guilty of manslaughter.^10^ Although initially welcomed by the judiciary, the 1922 legislation quickly proved problematic, with calls for further reform during the inter-war years. In expanding the remit of infanticide to include instances where a mother killed an infant under the age of twelve months while having failed to recover mentally from the ‘effect of giving birth or…the effect of lactation’,^11^ the 1938 Infanticide Act targeted a much broader group of female offenders. The Act remains in force today, with more than twenty nations having adopted their own legislation based upon the earlier British examples.^12^

Considering the influence British infanticide legislation has had globally and the explicit link inscribed in the 1938 Act between gender, offending, and criminal responsibility, it is unsurprising that legal scholars have devoted significant energy to its interpretation and implications.^13^ Indeed, Hilary Allen has claimed that infanticide legislation ‘seem[s] to promise an illustration par excellence of the psychiatrization of female crime’.^14^ Given the rules of diminished responsibility established under the Homicide Act 1957, scholars have also questioned the value of maintaining a separate offence/defence of infanticide.^15^ For those in favour of keeping the legislation, it is argued that it positively benefits women by marking them out as a group deserving of ‘special protection’ under the law,^16^ while Karen Brennan and Emma Milne have noted that in practice, the legislation ‘allows for “covert recognition” of social causes’ and ‘facilitates lenient treatment of at least some…women who kill their babies’.^17^

Given such complex responses to infanticide law, it is surprising that the intricacies surrounding the genesis of the 1938 Act have received relatively limited scholarly attention compared with the more developed historiography on reform attempts in the lead-up to the 1922 Act.^18^ By and large, the necessity of the 1938 legislation has been understood from a legal perspective, with the ongoing challenges the 1922 Act posed to the authority of the judiciary and legitimacy of the sentencing process, a central theme in this literature.^19^ This interpretation has notably been advanced by Tony Ward, who argues that although the 1938 Act deploys a ‘medical footing’^20^ in the language and reasoning it inscribes, this should not be seen as evidence of the medicalisation of the criminal law.^21^ Rather, ‘the infanticide acts involved a *reconstruction* of medical concepts to fit the needs of the law’^22^ at a time in which British psychiatry was already doubting the existence of a causal link between reproduction and maternal mental illness.^23^ Kirsten Johnson Kramar and William D. Watson provided partial revision of Ward’s thesis in their historical survey of psychiatric discourses on infanticide in Britain.^24^ Their analysis underscored ‘the entanglement of law, lay biological and moral reasoning, and a diverse range of formal bio-social psychiatric theories’^25^ in the context surrounding the 1938 Act. Although broadly supportive of Ward’s conclusions, in providing a commentary on the medicalisation of the law within the Act, Kramar and Watson have observed, that: ‘The mere fact that a bio-psychiatric interpretation of the causation of infanticide is written into documents as culturally significant as the English Acts, especially given that biological psychiatric thinking has gained extraordinary dominance within medical psychiatry in the last few decades, may be of some social significance’.^26^ Karen Brennan has also explored the medical rationale for the Infanticide Acts in her work. While noting that ‘medical experts seem to have [had] some input into the drafting of the Infanticide Act 1938’, Brennan has concluded that the parliamentary debates surrounding both Acts ‘reveal little concern with the medical validity of what was being proposed’ and suggested that ‘there appears to have been a lack of interest in the views of the medical community’.^27^Although recent work by Rachel Dixon has, for example, drawn attention to medico-legal debates on infanticide in the years between the Acts, further detailed analysis of the medical profession’s role in the creation of and response to the 1938 Act is wanting.^28^

This is an important consideration given that medical practitioners have historically played a central role in child-killing prosecutions and in campaigns to address infant mortality.^29^ Indeed, since 1822, expert testimony had been deployed in British courts to support the idea that some mothers harmed their children while suffering from discrete mental disorders caused by pregnancy, childbirth, or breastfeeding.^30^ As a ‘sudden, serious, but temporary, condition which could be cured either at home or in an institutional setting’^31^ doctors could explain the killing of infants by their mothers shortly after birth, or in the first months following delivery, to puerperal insanity,^32^ while married women who destroyed their babies several months after ‘their health had been broken down by continued efforts to breastfeed’ could be diagnosed as suffering from lactational insanity.^33^ As Hilary Marland has shown, the merit of these labels lay in their flexibility, explaining violent homicidal and suicidal behaviours that were out of character for mothers otherwise described as good and loving.^34^ The evidence of medical experts, especially psychiatrists, has remained integral to the practical implementation of the Act, and in light of changes in medical thinking on maternal mental illness, have also contributed to recent debates over maintaining the legislation.^35^ The contested relationship between reproductive states and mental disturbance that circulated in the lead-up to the 1938 Act, still persists, and was notably highlighted in the 2006 Law Commission Report on *Murder, Manslaughter and Infanticide.* ^36^ Despite acknowledging the disputed psychiatric grounds for maintaining the law, the Commission ultimately recommended maintaining the offence/defence of infanticide without amendment, noting that ‘the incidence of certain disorders is higher following childbirth. This temporal connection indicates that some women are more vulnerable to psychiatric disorder in the postpartum period’.^37^

This article analyses the genesis of the 1938 Act within key medical and legal discourses circulating in inter-war Britain. Situated against public debates over contraception and family planning, as well as the extensive media reporting of child-killing cases in the inter-war years, this paper underscores the vital role of medical practitioners in raising public and professional awareness of the necessity for infanticide reform. Crucially, it also assesses Dawson’s distinct contribution to the development and successful adoption of the 1937 Bill into law. Unearthing Dawson’s discrete contribution to this history is difficult. Surviving archival collections, personal correspondence, and biographical material on Dawson held at the Wellcome Library, Royal College of Physicians and elsewhere, are piecemeal, offering little direct insight into his views on infanticide or the inside story on how this Bill developed. Such methodological challenges partly explain why Dawson emerges as a neglected figure within the history of infanticide reform,^38^ and within inter-war British medicine, more broadly.^39^ Nevertheless, interrogating his published medical writings, personal contributions to parliamentary debates, media accounts of his expert testimony and involvement in inter-war medical campaigns, and government files, give insight into his motivations for reform and the steps he undertook to bring the Bill to fruition.

Following a concise overview of the development of the first Infanticide Act, this article surveys the response of medicine, law, and the press, to the 1922 legislation. Drawing upon an array of journal articles, published debates, correspondence, and case reports, it then analyses the durability of the connection between motherhood and mental states in shaping views on infanticide in inter-war Britain. Influenced by an active and broadly sympathetic press that reported extensively on infanticide, and the rising input of professional women to debates on law and motherhood, the article also investigates how this broad context paved the way for the implementation and favourable endorsement of Dawson’s measures by 1938. Turning next to the Hale trial and the legal and political responses it generated, the discussion then centres upon Dawson’s justifications for introducing his Bill and the actions he undertook to solicit medical and legal support for reform. Framed against the physician’s background and medico-political interests, it can be argued that the Bill embodied Dawson’s concern with preserving the respectable inter-war family amidst projections of decline in the quality and size of the population on the eve of war. This article closes by analysing reactions to Dawson’s reforms from the government, press, and medical and legal communities. In so doing, it raises questions surrounding the extent to which the legislation embodied the will of the broader medical community and whether it masked, rather than exposed, the very real lack of consensus existing within the profession over the relationship between reproduction, mental disorders and crime.

## The path to the Infanticide Act, 1922

Despite the inroads made by feminists and birth-control campaigners in the early decades of the twentieth century, most women in inter-war Britain had limited control over their reproductive lives, with little knowledge and access to birth-control. Although the increasing presence of contraceptives on British highstreets signalled an ‘increasing liberation of sexual attitudes’ in the aftermath of the Great War, such permissiveness was countered by a fear that the display, easy sale, and use of contraceptives might have a corrupting influence upon adolescents and the unmarried.^40^ Discourses on birth control were frequently suffused with eugenic ideas, whereby contraceptives were to be deployed not only for the benefit of the individual but for the nation as a whole. Jane Carey’s work has shown that the movement in inter-war Britain, the United States, and Australia, was deeply concerned with the ‘fitness’ of some groups to procreate and parent: ‘allowing “fit” mothers to have fewer but better babies, and decreasing the “degenerate” children produced by “poor” mothers’.^41^ Indeed, in Marie Stopes’ 1927 edition of *Birth Control*, she advised that contraception was medically indicated in families in which one or more of the parents struggled with alcoholism, poverty, feeble-mindedness, or puerperal insanity.^42^ Women who found themselves facing an unwanted pregnancy or unmarried and without support from the child’s father faced significant challenges, especially in an era in which sexual chastity remained closely tied to respectability and good womanhood.^43^

While the Infant Life Preservation Act, 1929, sanctioned abortion in instances where it was ‘done in good faith for the purpose only of preserving the life of the mother’^44^ in practice, the law remained highly ambiguous, exposing medical practitioners to the risk of criminal prosecution.^45^ Some women turned to dangerous and illegal procedures as a solution to an unwanted pregnancy, despite the significant risks such operations posed. The abstruse legal and ethical status of abortion prompted heated debates within medical and legal circles throughout the 1920s and 1930s. As with birth control, such debates were closely entangled with broader concerns over eugenics, population decline, and the fitness of the British ‘race’ during the inter-war years.^46^ Women who proceeded with their pregnancies faced the dangers of childbirth in a time in which maternal mortality remained high, despite infant mortality rates tumbling.^47^ Poor childcare provision and limited state financial support compounded the long-standing social stigma associated with unmarried motherhood and a woman’s survival as a single parent.^48^ Alcohol, domestic violence, and economic precarity also contributed to taxing home lives for many women. Married mothers, facing an unwanted pregnancy while struggling to raise older children against a backdrop of deprivation, had their helplessness compounded by financial dependence on their husbands. For some women, concealing their pregnancies and destroying their infants at birth (or soon after) presented a means to avoid community opprobrium, shame, and the loss of social or economic status.^49^

Eager to understand and extend sympathy towards such women, jurors exercised discretion in criminal proceedings. This could include returning a verdict of insanity based on limited evidence, downgrading capital murder to the lesser offence of concealment of pregnancy (where the victim was a newborn), or recommending mercy on compassionate grounds.^50^ Judges also used their sentencing remarks, widely reported in the press, to cast responsibility on the fathers of illegitimate children who rarely faced prosecution. As Ward has noted, the discourse of the ‘abandoned, impoverished, but ostensibly good’ ‘fallen woman’ encouraged sympathy and lenient treatment of women accused of infanticide.^51^ Nevertheless, prior to the legislative changes of the twentieth century, English and Welsh women convicted of the murder of their newborn infants were sentenced to death, just as any other defendant found guilty of murder. From 1849 onwards, such women were never hanged; the death penalty was instead commuted.^52^ Judges nonetheless observed the formality of the law by donning the ceremonial black cap and pronouncing the death sentence in court. From the early decades of the twentieth century, the label ‘black cap farce’ was applied to this incongruous spectacle of judicial sentencing.^53^ Pronouncing execution on mothers while knowing the sentence was never to be carried out was seen as an unnecessary cruelty against women who had already suffered. Consequently, a significant gap emerged between the letter of the law and its application.

Conflicting views among politicians and policymakers, together with concerns that significant change to the law on homicide might imply that child-killing was not as serious an offence as murder, led to repeated failures to reform the law in the 1860s, 1870s, and 1900s.^54^ However, as Daniel Grey and Anne Logan have shown, following the groundswell of popular outrage against the capital sentence imposed on Edith Roberts for the murder of her newborn daughter in June 1921, female magistrates deployed their professional networks and political connections to agitate in the press, and pressure the Home Office (HO) to make legislative changes.^55^ Subsequently, Labour MP Arthur Henderson introduced a bill into the House of Commons that attempted to reform the law on child murder.^56^ Despite the Bill being rejected, policymakers proceeded to draft and implement their own legislation.^57^ On 20 July 1922, the first Infanticide Act was granted royal assent. A woman could be convicted of the new offence of infanticide if she was found responsible for the killing of her ‘newly-born child’ while ‘the balance of her mind was…disturbed’, having failed to recover ‘from the effect of giving birth’.^58^ Such women were subsequently ‘dealt with and punished’ as if they had committed manslaughter. Emma Temple was the first woman to be tried under the new Act, at Lincolnshire Assizes in October 1922. Having pled guilty to the infanticide of her newborn daughter, Mr Justice Lush then sentenced Temple to four months’ imprisonment and commended the new Act as ‘a wise and humane piece of legislation’ that made it unnecessary ‘to put a girl on trial for murder’.^59^

## Responses to the Infanticide Act, 1922

Positive reception to the first Infanticide Act did not last long, with the language and practical implementation of the legislation proving a stumbling block for police, coroners, lawyers and judges. Describing the 1922 Act as ‘beneficent but badly drafted’, Ernest Wild, Recorder at the Old Bailey, lamented in January 1925, that: ‘the grand jury could do nothing where there was a charge of murder but bring in a bill for murder and leave the petty jury to find a verdict of infanticide’.^60^ As a result, women suspected of the lesser offence of infanticide were still being marked with the stigmatising label of ‘murderess’. Arriving at the appropriate sentence for women convicted of infanticide was also problematic. On 23 November 1925, an appeal against the sentence imposed on Florence Irene Chick was heard at the Court of Criminal Appeal.^61^ Chick had been convicted of infanticide and concealment of birth in October and sentenced to nine months’ imprisonment. She appealed the judgement on multiple grounds, with her counsel also arguing that she had received a more punitive tariff than a defendant in a similar case tried at Liverpool Assizes around the same time. Although acknowledging that ‘it was a serious offence’, the appeal judges reduced her sentence to three months’ imprisonment in the second-division.^62^ Nevertheless, the HO maintained that the legislation had achieved its intended goals, with an internal analysis of prosecutions for the five-year period ending 1927, concluding that: ‘some women who…would have been convicted only of concealment of birth, have since 1923 been convicted of infanticide – which make the operation of law correspond more clearly with facts’ and ‘that several have been convicted of infanticide who would formerly have been convicted of murder’.^63^

Nonetheless, the 1922 Act continued to receive a cool reception in courtrooms and from the medico-legal community, with criticism centring upon how to define and interpret the phrase ‘newly-born child’. The issue reached a critical juncture in the case of Mary O’Donoghue, who had been convicted of the murder of her 35-day-old infant at the Old Bailey in October 1927, before Mr Justice Talbot. Her counsel, in appealing her conviction less than a month later, argued her deceased infant could be deemed ‘newly-born’ within the provisions of the legislation and that the jury should have been allowed to consider a verdict of infanticide, given ‘such a question [had been left] to the jury in the case of a child of six weeks old’ tried at Reading Assizes in October 1923.^64^ The appeal judges rejected these arguments.^65^ In January 1928, the editors of the *Journal of Mental Science* suggested that the judgement had done little to settle the matter of what the phrase ‘newly-born child’ meant and that ‘criticisms will not be diminished in force by the occurrence of the present case’.^66^

This editorial was revealed to be prophetic when only one month later, Dr Ann Louise McIlroy gave an address on infanticide reform before the London Medico-Legal Society.^67^ McIlroy was an influential female doctor and the first woman to be appointed professor of gynaecology and obstetrics at the Royal Free Hospital in London. She took a keen interest in maternal health, birth control, and reproduction and wrote at length on the responsibilities of the medical profession in these areas.^68^ McIlroy contended that while the Act offered some protection to women who killed their babies immediately after childbirth, it stigmatised mothers suffering from other forms of mental illness who killed while disturbed from pregnancy, the puerperium or breastfeeding.^69^ Under the terms of the McNaghten rules, McIlroy feared a woman suffering from puerperal insanity but who ‘murdered her child knowing that she was doing wrong’ might be convicted of murder and consequently stigmatised and punished by an extended period of incarceration.^70^ Given ‘the law absolves the mother who commits infanticide upon her ten-day-old child’, McIlroy argued ‘it should just as readily absolve the depressed mother, who, worn out by lactation, puts an end to her infant’s life’.^71^ McIlroy recommended removing the phrase ‘newly-born’ from the existing Act and extending its operation to mothers who killed their infants in the six to nine month period after childbirth.^72^ Such provisions were not only medically sound, but also upheld the responsibility the British state owed its female citizens to be merciful and compassionate, after all: ‘Motherhood is a sacred and patriotic service, and no woman should run the risk of being branded a murderess because she happens to fall a victim to one of the diseases incident to pregnancy’.^73^

McIlroy’s proposals and her broader discussion of infanticide reform met with a varied reception from the Medico-Legal Society. Although members agreed that the language of the legislation was problematic, there was no consensus on how the ‘newly-born question’ should be settled. Criminal court judge, Mr Justice Humphreys, admitted: ‘The Act was probably unnecessary’ but felt its retention helped ‘prevent the horrible farce of a judged assuming the black cap and sentencing to death a person who…was not in the last danger of being hanged and who would probably be released in few weeks’.^74^ Marie Stopes and Medical Superintendent of Springfield Mental Hospital, Dr Reginald Worth, agreed that the Act should be extended and cover the murder of infants not yet weaned.^75^ Other Society members felt a more restrictive period in which a child was defined as ‘newly born’ should be imposed by the legislation.^76^

The debate also centred on the broader questions of what (if any) role reproductive mental disorders played in infanticide and how to define the mental disorders that followed pregnancy, birth, and breastfeeding. In her paper, McIlroy argued that puerperal insanity was a ‘term used to denote an abnormal state during pregnancy, labour, the puerperium or lactation’ and could be broken down into three forms: ‘confusional, intermittent (manic-depressive), and dementia praecox’.^77^ Ten days following childbirth, women were ‘more likely to be melancholic, suicidal, and homicidal towards the infant’.^78^ Meanwhile, ‘badly nourished, overworked and poorly fed women’, worn down by the physical and mental strain of suckling an older infant, were more likely to be ‘melancholic in type’ and at greater risk of killing ‘on impulse’ than those who killed an infant in the days after its birth.^79^ The association McIlroy identified between reproductive states and mental maladies was echoed in leading British texts on women’s health. The 1928 edition of the *Combined Textbook of Obstetrics and Gynaecology* remarked that pregnancy and the puerperium could trigger disturbances in the nervous systems of women, leading to insanity, especially when complicated by prolonged labour and lactation, sepsis, exhaustion and a previous (or familial) history of mental illness.^80^

Reflective of British psychiatry’s mounting scepticism of a definite causal association between childbirth, lactation, and insanity, Dr Hubert Bond challenged McIlroy’s assertion that women killed while affected by distinct reproductive insanities.^81^ In a savage critique, Bond asserted that: ‘there is no mental disorder…which, by its mental symptoms and in the absence of knowledge otherwise gained, can be recognised and diagnosed as due to child-bearing’.^82^ He also claimed that maintaining ‘the use of such adjectival terms as puerperal, lactation’ to describe a woman’s mental condition created confusion by suggesting that such disorders existed distinctly in their own right.^83^ Dr Norwood East, an inter-war expert on male psychopathology, had also abandoned the use of ‘puerperal’ and ‘lactational’ insanity in his textbook *An Introduction to Forensic Psychiatry^84^.* In response to McIlroy’s paper, he suggested that the Act afforded greater consideration to the plight of women than of men, who also killed children in distressing circumstances. East opined that reform was unnecessary and that ‘although the law was illogical, in practice justice was done’.^85^ In the face of these critiques, McIlroy doubled down on the sex-specific distinctiveness of reproductive mental disorders, concluding that: ‘Childbirth was so great an ordeal, even to the most normal woman, that its effects on the mind could not be classed with any other form of mental disorder’.^86^ Although McIlroy’s paper and responses to it were disseminated to a broader professional audience via the *British Medical Journal* and *The Lancet*, it did not prompt any initiatives to reform the law.^87^

## Medical and legal developments, 1930 - 1935

Incremental improvements in the legal treatment of mothers, however, were introduced during the early 1930s. The Sentence of Death (Expectant Mothers) Act was passed in the 1931 Parliament in direct response to the capital sentence imposed on 37-year-old pregnant mother Olive Kathleen Wise for the murder of her 9-month-old son.^88^ This legislation abolished the recording and pronouncement of the death penalty on pregnant women, replacing this sentence with penal servitude for life.^89^ In Autumn 1931, two Middlesborough MPs, Ellen Wilkinson and Frank Kingsley Griffiths, lobbied the Home Secretary for the release from Durham Gaol of 43-year-old mother, Catherine Cunningham.^90^ Cunningham had been sentenced to three months imprisonment for attempted suicide by stipendiary magistrate Mr H.S. Mundahl in September 1931. As a respectable mother of fourteen children who had attempted to gas herself while plagued by significant financial and personal worries, Cunningham’s sentence prompted public outrage.^91^ Following pressure from politicians, social workers, and the press, ten days after sentencing, she was liberated, on order of the Home Secretary.^92^

Meanwhile, the medical profession continued to ruminate over the potential causal link between insanity and female reproductive states. Certainly, some in the psychiatric profession pronounced the death of puerperal insanity and its related disorders with the same confidence that Hubert Bond had evinced in 1928. Dr E.W. Anderson, Assistant Medical-Officer at the Maudsley Hospital, opened his 1933 paper ‘A Study of the Sexual Life in Psychoses Associated with Childbirth’ with the declaration that: ‘It is now universally recognized that there is nothing specific in the form of psychoses associated with childbearing’.^93^ However, when the opinions of the psychiatric profession were canvassed more broadly, there was less consensus on the subject.

That same year, the *British Journal of Gynaecology* shared the findings of Dr Arthur Leyland Robinson’s survey of the attitudes of British alienists on the effects of reproduction upon insanity.^94^ As Professor of Midwifery and Gynaecology at the University of Liverpool and Honorary-Surgeon at Liverpool’s major maternity hospitals, Robinson was firmly entrenched in inter-war obstetric thinking and practice. In sympathy with many alienists, Robinson endorsed the belief that: ‘No specific kind of mental disturbance is produced by the physical and psychical changes that follow conception’.^95^ However, Robinson was reluctant to deny a causal association between reproduction and insanity, noting that ‘the results of conception may have a specific effect upon the disordered mind. This latter view has long been the cherished belief of the obstetrician by whom mental and nervous changes of early pregnancy have been regarded as characteristic and indeed pathognomic’.^96^ Curious to canvas the views of British psychiatrists, Robinson surveyed ninety-five alienists on a variety of questions, including what opinion mental specialists held as to ‘the effect of the process of reproduction upon the incidence of insanity’.^97^ The results of the survey revealed that the majority of alienists did not think that ‘child-bearing is… a…specific cause of insanity, but may act as an exciting or contributory factor in the presence of hereditary predisposition and physical or mental stress’.^98^ Only thirteen percent of those evaluated felt that childbirth caused insanity outright, while the same percentage of participants felt it had no influence whatsoever.^99^ Given that almost seventy percent of surveyed mental specialists felt that there was some link between childbirth and insanity, Robinson’s study demonstrated that few psychiatric practitioners were as adamant as Anderson in denying a potential causal link between reproduction and mental illness. It was evident, too, that both specialties shared a belief that even if childbirth did not produce distinctive forms of maternal mental disorder, reproductive states nevertheless had an important impact upon the mental health and psyche of women.

Against such a backdrop, an accommodating space for medical practitioners to champion a variety of sympathetic explanations for maternal child-killing was able to flourish. For example, in September 1935, *The Manchester Guardian* reported on Dr Edith Summerskill’s campaign to reform the criminal justice system’s response to insane mothers.^100^ Summerskill, a self-proclaimed socialist and feminist who won the seat of West Fulham for Labour in 1938, campaigned in the areas of maternal health, birth control, and welfare provision throughout her career. Observing the living conditions of her poorer patients since her early days in practice, she argued a direct causal link could be drawn between motherhood, insanity and the hardship endured by working families impacted by the Depression: ‘The question of puerperal insanity is rather a terrible one…The mothers, when their husbands are out of work, have such a time half-starved and worried by the extra addition to the family, and this all serves to aggravate their mental condition’.^101^ Summerskill conveyed her hopes to the *Manchester Guardian* that Parliament might consider introducing a measure where mothers who murdered their children, while suffering from puerperal insanity, might be assessed by ‘a committee of alienists and psychiatrists’ and treated medically, rather than be subjected to the ordeal of committal and trial at police courts or assizes. However, her proposals gained little traction beyond some brief press interest.

## Lord Dawson of Penn and *R v. Hale* (1936)

Almost nine months later, on 21 July 1936, Brenda Hale was brought to trial before Mr Justice Travers Humphreys at the Old Bailey.^102^ Hale’s lawyers attempted to argue prior to her trial that her case should be prosecuted under the remit of the Infanticide Act 1922 rather than a capital murder charge.^103^ This was based on the fact that the infant was twenty-two days old at the time of its death and that no timeframe had been defined by the 1922 Act in which a child was (or was not) ‘newly-born’. If successful, it was hoped she could avoid the imposition of a ‘Guilty, but Insane’ verdict and the subsequent incarceration at a mental institution (for an indefinite period) that this would entail.^104^ Alongside Hale’s attending obstetrician, Dr Medlock, Lord Dawson of Penn was also called as an expert witness in support of the defence argument.

While no records survive explaining why Hale’s legal team specifically sought Dawson’s opinion as a medical witness, a brief review of Dawson’s professional resume offers ample evidence of his expert status. Sir Henry Bashford’s obituary to Dawson, published in *The Spectator* in March 1945, described him as ‘the most widely known physician in the English-speaking world. He was also one of the most trusted’.^105^ At the time of the Hale case, Dawson was in his early seventies, having reached the pinnacle of a long and successful career in the public eye. He practised as a consulting physician in Harley Street until 1940 while also maintaining a long-running affiliation with The London, where he had completed his residency and gained his earliest institutional appointments.^106^ In 1907, he was appointed physician-extraordinary to King Edward VII and served in the Medical Households of successive monarchs as a royal physician.^107^ When Dawson was made a Baron in 1920, he became the first practicing member of the medical profession to be elevated to the peerage in the House of Lords, and in the same year as the Hale trial, he was made a viscount.^108^

Dawson’s involvement in numerous governmental advisory bodies, medical organisations and inter-professional committees extended his influence. He had an ongoing relationship with the Ministry of Health and played a significant role in advising upon the organisation of post-war medical services, culminating in the Dawson Report in 1920.^109^ During the inter-war years, he was president of the Medical Society of London, the British Medical Association (BMA), and the Royal College of Physicians (RCP). He was also involved in inter-professional committees and networks which garnered press attention, including the Medico-Legal Society and Advisory Committee on the Scientific Detection of Crime.^110^ Significantly, Dawson used these appointments to advocate for greater involvement of the medical profession in service to the state. His personal philosophy was embedded in an idea of citizenship in which the nation benefited most when the individual and state actively and mutually served each other: ‘What we seek is men…to solve the problems of life and health…For citizenship should be a recognition of mutual dependence and the value of service. The state claims for each citizen a positive allegiance, not a negative acceptance’.^111^

Dawson’s internationally disseminated speech to the Church Congress at Birmingham in October 1921 advocated the use of birth control within marriage and endorsed sex as important to this union.^112^ For Dawson, birth control offered an efficacious way to manage the expansion of respectable English families so that a mother would not be worn down by the physical and emotional stress of successive births and childrearing.^113^ Into the 1930s, he maintained that the ideal family should have at least three children to support not only the ‘happiness and enduring youth’ of the individual but also so that British imperial sovereignty would be guaranteed for successive generations.^114^ In support of this mission, Dawson championed schemes to improve the quantity and quality of the British populace amid reports of a low birth rate and apocalyptic projections of population decline.^115^ In addressing the York Medical Society in November 1935, he argued for improvements in nutritious food provision and physical fitness for the young, as well as milk subsidies for children, pregnant women, and nursing mothers.^116^ Dawson also endorsed negative eugenic schemes, including segregation and the voluntary sterilisation of ‘mental defectives’, by arguing that society could not ‘afford to have these vicious strains breeding in our midst’.^117^ The physician’s pro-natalist standpoint was well-acknowledged, if not universally endorsed,^118^ with *Punch* satirising his campaigning for the Physical Training and Marriage Bills in mid-1937 under the captioned cartoon of ‘A Brace of Babies’.^119^

At the Hale trial, Dawson’s testimony on the ‘newly-born question’ was deployed as part of the broader defence stratagem to bring the case within the remit of the 1922 Act. Dawson noted that the phrase had ‘never been defined in medicine’^120^ but endorsed the practice adopted by medical statisticians and the Ministry of Health, who included ‘any child …up to four weeks old’ as ‘newly born’.^121^ Despite this strategy, Humphreys J asserted that the Hale case was beyond the provisions of the Act as he was bound by the O’Donoghue decision. Hale’s team were more successful, however, in arguing that she had failed to recover from the effects of giving birth and so was not legally responsible. Following Dr Medlock’s evidence of Hale’s medical history and disturbed mental state following the crime, Dawson testified that:

[H]e thought Mrs Hale was suffering from puerperal insanity when she killed her child, and he agreed with Dr Medlock that she would not know what she was doing.

Mr Hutchinson: How long would you say a woman remains affected by childbirth?

– I should say not less than three weeks.

His Lordship: Does that apply to all women?

– To all women of civilised races.^122^

Humphreys J stated during the proceedings that the lack of legal clarity over the phrase ‘newly-born child’ remained deeply problematic. However, Dawson’s definition would be helpful to legislators, with Humphreys J expressing the ‘hope that Parliament might…put a short section into the Act…saying what is the definition of a newly-born child’.^123^ In his remarks to the jury, the judge also conveyed his frustrations with the language adopted in English criminal law to describe an acquittal on the grounds of insanity, arguing that a ‘Guilty, but Insane’ verdict erroneously implied that a defendant was guilty of murder.^124^ Evidently persuaded by the case advanced by defence medical experts and the opinions of the presiding judge, the jury returned the novel verdict of: ‘Not guilty of murder but guilty of the act charged for which she was not responsible in law’. Hale was ordered to be detained for His Majesty’s Pleasure.

## Dawson’s Infanticide Bill

The Hale trial prompted a strong press, and political and public reaction. In reporting the case, the English press had been quick to note that the trial was one of three maternal child-killing cases before Humphreys J at the Central Criminal Court that week alone.^125^ For example, the *Daily Herald* reported the outcome of the Hale trial alongside updates on the cases of Helen Alcorn and Gweneth Enid Dryland, two married English women who had also killed their own children. In a central page spread that included a photograph of 24-year-old Alcorn posing lovingly with her (since deceased) infant daughter, the paper reminded its readers of the emotional damage and strain the verdict, and its aftermath, wrought upon the families of the tried women. Alcorn’s husband confided to the *Daily Herald* that: ‘It was a terrible ordeal to look forward to with the sentence hanging over her’.^126^ The arbitrary nature of the law also became a subject of parliamentary debate. On 28 July 1936, Rhys Davies, Labour MP for Westhoughton, asked the Secretary of State during question time in the House of Commons why Hale had not been prosecuted under the 1922 Act, given ‘the facts showed that this was a case for which the Infanticide Act was intended’.^127^ Officials in the HO and Lord Chancellor’s Office (LCO) had been monitoring the outcry and ill-fated attempts to reform the 1922 Act since the start of 1936.^128^ The National Council for the Abolition of the Death Penalty (NCADP) had been particularly active in lobbying sympathetic MPs to amend the offence in the eighteen months prior to the Hale case.^129^ Conservative MP for Wallsend, Irene Ward, had corresponded with the HO on an Infanticide Bill drafted under the NCADP’s recommendations. The Bill proposed extending the offence to mothers who killed children up to the age of eight while suffering from a disturbance of mind following childbirth, or from ‘distress and despair arising from solicitude for her child or extreme poverty or other causes’.^130^ Ward’s proposal was rejected by the Home Secretary, Sir John Simon, who emphasised his reluctance to consider reform. Labour MP, John Jagger, introduced a bill into the House of Commons modelled on the same parameters as Ward’s proposal four months after the Hale case.^131^ It suffered the same fate as Ward’s measure.^132^

Pressure had also been mounting on the government from two of the principal actors in the Hale case. One day after Humphreys J had presided over the proceedings at the Central Criminal Court, he wrote a two-page letter to the Lord Chancellor on the renewed issues the Hale case posed for the judiciary. Specifically, he raised the question of whether the phrase ‘newly-born child’ might be settled through the introduction of a bill in Parliament.^133^ Schuster replied on behalf of the Lord Chancellor, noting that when the 1922 Bill was drafted, it had been intended that the ‘newly-born question’ would ‘work itself out’^134^ and that ‘the Judge would leave it to the jury’ to decide.^135^ Schuster failed to acknowledge in his reply that he had been intimately involved in the drafting of the Act and was responsible for the adoption of the problematic phrase.^136^ He admitted, however that the Infanticide Act had never been intended as a measure to include mothers who killed older infants, or where there was clear evidence of mental insanity, as in the case of *R v. Hale*: ‘the statute was intended to apply where…the woman might be…in the state of physical and mental confusion…as usually comes…in these cases’.^137^ Records demonstrate that Humphreys also conferred in-person with Alexander Maxwell, Deputy Under-Secretary of State at the HO, on amending the legislation. However, as with Schuster before him, Maxwell gave little practical support to Humphrey’s recommendations. Thus, despite the efforts of the High Court judge, penal reformers and sympathetic MPs, by November 1936, it was evident that the various government ministries entangled in the question of infanticide reform were ‘in favour of doing nothing’^138^ to change the law.

By Autumn 1936, a separate campaign for reform was progressing under Dawson’s ministrations. In correspondence to the LCO, Humphreys J noted that he and Dawson had discussed the ‘newly-born question’ following the Hale case.^139^ In this exchange, Dawson had confided that ‘a definition would be welcomed by the doctors…He would support a month in the House of Lords’.^140^ According to the judge, Dawson ‘ha[d] always been interested in this problem’^141^ and it is likely that the shared involvement of the two men in the Medico-Legal Society further enabled Humphreys J to solicit his opinion. Infanticide reform certainly harmonised with Dawson’s interest in maternal welfare at the time of the Hale case. At a dinner of the British College of Obstetricians and Gynaecologists three months after the trial, he conveyed the hope that public anxieties over the safety of childbirth might be alleviated if ‘we get it into the mind of the people that maternity is a safe proceeding and that the risks…are…far less than in many common events of daily life’.^142^ Although it remains unclear at what point interest in the problem transformed into an active campaign, by September 1936, Dawson had written to Edward Hale Tindal Atkinson, Director of Public Prosecutions (DPP), requesting ‘statistics of infanticide…passing through this Department from the beginning of 1935’.^143^

Records also indicate that Dawson drafted his Bill based on consultation with medical and legal practitioners. As President of the RCP, he acknowledged canvassing members for their advice on the legislation, referring the question of infanticide reform to ‘a committee of experts’.^144^ Unfortunately, the recommendations of the RCP’s committee, opinions of ‘leading King’s Counsel’, and the ‘considerable correspondence’ Dawson received on infanticide reform do not appear to have survived.^145^ However, a paper delivered by Dr J.C.M. Matheson to the Medico-Legal Society in April 1941 suggests that Dawson may have sought the opinion of prison medical officers as part of his consultation within the medical profession.^146^ Crucially, as Governor and Medical-Officer of Holloway Prison in 1936, Matheson reported on the mental fitness of Hale for trial, and so was intimately connected with the case.^147^ As Stephen Watson has shown, prison medical officers were ideally placed to speak as experts on questions of mental responsibility due to their proximity to and observation of prison populations.^148^ Dawson also used his networks within psychiatry to gather data upon which to justify the parameters of his Bill. Indeed, HO correspondence indicates that Dr A.E.W. Petrie, Medical Superintendent at Banstead Hospital and Lecturer in Psychological Medicine at Charing Cross Hospital, was working with Dawson to support the implementation of his Infanticide Bill. Writing to Dr Foulerton, Deputy-Medical-Officer at Holloway Prison in March 1938, Petrie noted: ‘Dawson is very anxious to obtain further confirmation of the necessity to legislate for those after the immediate puerperium, ie. lactational cases…Lord Dawson was much impressed with your figures and those of Dr Hopwood…Can you or Dr Hopwood help?’^149^ Dawson’s ability to deploy professional connections in service of infanticide reform underscores not only his significant practical influence within the profession but also demonstrates how he believed medicine could fruitfully serve the state in helping to ‘solve the problems of life and health’. That legal and medical practitioners were evidently willing to back Dawson’s, cause reflects an eagerness to reach a point of working clarity on the question of infanticide.

Just before Christmas 1937, Dawson introduced his Infanticide Bill into the House of Lords. The private member’s bill was positively touted in the British press as ‘Mercy For Mothers’^150^ allowing a woman who killed her infant, aged less than one year old, to be found guilty of infanticide if she committed the crime while the balance of her mind was disturbed following the child’s birth or from the effects of lactation. As with the 1922 Act, a convicted woman would be dealt with as though she had committed manslaughter. Although more conservative than the reforms proposed by Summerskill, Ward, or Jagger, Dawson consciously portrayed the Bill as adopting ‘the middle course’ within the spectrum of options open to reformers. As he observed, this approach was intended to be a measured and sympathetic solution to the infanticide question, for: ‘in the case of a woman who has, after all, done a great public service in bringing children into the world, and who goes through a time of great stress, especially in these days, I think we are entitled to treat her better than the law does at present’.^151^

Between the Bill’s First Reading and its eventual passage into law as the second Infanticide Act in June 1938, Dawson used his speeches in the Lords to win support for his reforms. Leveraging his professional experience, networks, and status as a physician, Dawson framed infanticide reform as primarily a medical concern, justifying his leadership of the campaign. In the Second Reading of the Bill on 22 March 1938, Dawson made his position and intentions clear:

[T]he intention of this Bill is to secure recognition by Parliament that under certain circumstances the killing of infants is provoked by illness and not always by criminal intent, and to procure for such cases appropriate handling. The subject matter of the Bill belongs to the territory where law and medicine meet.^152^

He also referenced his personal involvement in the infanticide question by citing his role as an expert defence witness in the Hale case and drawing upon the data he had acquired from Hopwood and Foulerton as to the prevalence of infanticidal mothers within Broadmoor Criminal Lunatic Asylum.^153^ For Dawson, mothers who killed while suffering from puerperal insanity or related conditions should be recognised in the criminal justice system as sick rather than bad women, and: ‘as much cases of illness as any other complications of child-birth, as much as the physical complications, quite as much as streptococcal septicaemia, quite as much as white lead…They bear perfectly clear evidence of temporary insanity’.^154^ In casting infanticide as a medical object, his rhetoric also worked to implicitly justify a continued space for the profession’s expertise in child-killing prosecutions in twentieth-century British courts.

Dawson was also keen to cast his Bill as a measured and sympathetic response to the suffering of respectable, loving mothers, whose subsequent exposure to the criminal justice system became a source of shame and trauma. Citing the Hale case anecdotally, Dawson noted that prior to the crime, the farmer’s wife had been: ‘sane; she was happily married, and a good mother’.^155^ When at trial a defence of infanticide had not been open to her, Dawson noted that the judge and jury were left with no choice but to find Hale ‘Guilty, but insane’ and so commit her to indefinite incarceration, thus: ‘these people have the alternative either of being convicted of murder, with all the slur that that involves to themselves and their families, or of being committed to Broadmoor, which leaves upon them the stamp of insanity’.^156^ During the Committee phase two weeks later, Dawson stressed once more the shattering impact infanticide wrought upon the domestic felicity of the inter-war family:

Take any happy home you like, perhaps that of a newly-married couple…and consider the additional happiness of an expected first-born child. Suddenly…the mother destroys her child. It is difficult to imagine anything which would cloud the skies of life more blackly than that.^157^

The adoption of such reforms would, Dawson argued, ‘remove once and for all a stigma about which public opinion has been disturbed for sixty years’,^158^ by better recognising in law the sometimes fatal link between motherhood, mental disorder, and violence. Dawson’s focus on the Hale case as providing both inspiration for, and an example of, the type of women that his reforms could help, signalled a shift away from the desperate, unmarried infanticidal mother of archetype, targeted by the earlier Act. Reshaping the object of his Infanticide Bill to this type of offender, not only created a distinct gap within the existing law for Dawson’s Bill to fill, but also helped channel pre-existing sympathies within the press, Parliament, and the broader public, to a recognisable and sympathetic embodiment of ‘respectable’ femininity and tragic motherhood. Although speculation, such women also upheld the kind of family-centric womanhood that Dawson saw as vital to the longevity of the British race, and which were doing their biological and national duty by ‘bringing children into the world’.

## Reactions to the Bill

Although Whitehall officials remained averse to reform, the appetite for legal change was described as strong in the British press and was broadly popular among parliamentarians. Atkinson remained steadfastly opposed to Dawson’s proposals, citing the risk that an unwanted child might be murdered ‘in circumstances in which a dishonest defence might be put forward inducing a verdict of infanticide’.^159^ Moreover, Dawson’s bid to abolish the verdict of ‘Guilty, but Insane’ in infanticide cases was abandoned when it was revealed that such changes were beyond the powers of his Bill and required wholesale changes to criminal procedure.^160^ Nevertheless, influential civil servants like Maxwell ultimately adopted a pragmatic view of the legislation:

I do not feel a case could be made out against the Bill on the ground that it increases the killing of unwanted children…popular feeling is much less moved by this consideration than…the spectacle of a mother being sentenced to death after she has killed the child and possibly attempted suicide in circumstances of great distress.^161^

In a letter dated 8 February 1938 to Maxwell, Schuster remarked that having shown the Bill to the Lord Chancellor: ‘He agrees that it is no use to oppose the Bill. He would, however, like the Home Office representative to sniff at the Bill in the House of Lords. It is obviously a very silly bill’.^162^

Unlike policymakers, the British press welcomed Dawson’s proposals. The *Daily Telegraph* reported glowingly on the Bill, noting of its Second Reading in March 1938, that: ‘The Socialist and Liberals, the Law Lords and the Primate joined, “in the interests of justice and pity,” with Viscount Dawson of Penn in furthering his Infanticide Bill’.^163^ The *Daily Express*, in describing the Bill under the headline ‘A Law of Mercy’, remarked that: ‘Whatever spares us the senseless cruelty of condemning poor mothers to death…while half-crazy with the pain of childbirth is GOOD’.^164^ Even sceptics, like conservative writer and columnist Gilbert Frankau, seemed to approve of Dawson’s ‘sincere and common-sensical measure’.^165^ Nevertheless, Frankau warned that further reforms, if left unchecked, could contribute to the broader dismantling of capital punishment for women.^166^

The response within the legal profession to Dawson’s proposals was generally positive, although questions remained over the legislation, particularly whether it was necessary or would be effective in practice. In January 1938, the *Law Journal* endorsed the Bill, stating that:

Every medical man, as well as most laymen, knows that the effects of childbirth…are…more lasting…than for a period of a few weeks. Lord Dawson would have the period fixed at a year…it seems a reasonable period. In such cases mercy is justice, and should not be denied.^167^

J.A. Palmer, writing in the *Justice of the Peace and Local Government Review*, was more cautious, querying the extent to which the time limits of the Bill might raise broader questions for the maintenance of capital punishment more generally.^168^ Although the *Medico-Legal Review* observed that the legislation ‘would considerably widen the class of woman…found guilty of infanticide’^169^ the editors questioned whether the Bill would appease those who wanted more radical reform, so that ‘a woman who was in truth irresponsible when she killed her child should be dealt with like any other mental patient and not treated as a “criminal lunatic”’.^170^ This view was also echoed separately by Palmer, who claimed that such a Bill should be unnecessary given that the criminal law recognised that the insane were not fit subjects for punishment.^171^

In January 1938, *The Lancet* endorsed the comments of female magistrate R.H. Crowley, who argued that in infanticide cases, ‘the medical aspect is too little considered’.^172^ Crowley had been horrified to witness ‘a young woman…apparently committed under the influence of puerperal insanity’ and ‘caged like some savage animal’ in a provincial prison while awaiting trial for infanticide.^173^ *The Lancet* endorsed Dawson’s Bill as a ‘desirable reform’ but hoped that: ‘Mrs Crowley’s point – namely, that the accused mother is a fitter subject for observation and treatment in a mental hospital than in a prison cell’ would be added to the reforms considered by the Lords.^174^ In May 1938, the Parliamentary Subcommittee of the BMA backed Dawson’s Bill while also proposing an amendment: ‘to provide that when a woman charged with infanticide was remanded or committed for trial she should be remanded to a mental hospital and not to prison’.^175^ Dawson attempted to enact the Subcommittee’s suggestion, raising the issue within parliamentary debates of his Bill. In HO records, it was determined some months later that although no procedural change could be implemented through the proposed Infanticide Bill, the Chief Magistrate agreed that local Justices should deploy their discretionary powers more fully to bail women to suitable places of security.^176^

## The Infanticide Act 1938

On 23 June 1938, Dawson’s Infanticide Bill was passed into law, with minor amendments, following a seamless passage through the House of Commons. Despite an extended period of gestation, the Infanticide Act 1938 received little immediate fanfare. Instead, medical, legal, and media attention redirected to the issue of abortion in the wake of the trial of gynaecologist, Dr Aleck Bourne, in July 1938.^177^ By the spring of 1939, debates within the Lords on the alarming failure of ‘the nation…to reproduce itself since 1925’^178^ captured Dawson’s attention once more. Family allowances, payable to mothers, and housing built with family life and childcare provision in mind, should be part of the solution, Dawson argued.^179^ However, impressing on women the psychological fulfilment motherhood offered and that reproduction was a national imperative to ensure ‘our people shall be adequate in numbers and stable in their civilisation’, was just as critical too.^180^

Despite infanticide law receding from public scrutiny, Dawson’s reforms had a palpable impact. In the ten years after the Act, the annual average figure of prosecutions for infanticide at assize and quarter sessions more than doubled.^181^ The first woman tried under the legislation was 29-year-old married mother, Jennie Margaret Dormer.^182^ Dormer had been depressed following childbirth and struggled to feed her 10-week-old daughter. Her mental condition had deteriorated further in response to the growing threat of conflict with Germany, following the May Crisis in Czechoslovakia. In October 1938, she fatally gassed her daughter. During her incarceration at Holloway, her mental health improved and following the acceptance of her plea of infanticide, Dormer was bound over for two years and released into the care of a friend. Subsequent trials across the country would see the provisions of the new Act endorsed by a judiciary eager to explain the violent actions of infanticidal mothers as proof of mental disorder. In October 1939, married 42-year-old mother Annie Slater was found guilty of infanticide and sentenced to two months’ imprisonment for the drowning of her son. Mr Justice Stable took pains to assure her that incarceration was not a punishment, but a chance to recover physical and mental health while in the prison hospital.^183^

Although Dawson described the 1938 Act as a collaborative expression of medical and legal opinion on the question of infanticide, the legislation did little to resolve the ambivalent attitude of the medical profession to the existence of distinct reproductive insanities. Thirteen years after McIlroy had raised the ‘newly-born question’ before the London Medico-Legal Society, the same debates over diagnosis and the legal response to mothers who killed, were played out once more. Matheson, who had been involved in the Hale case and (likely) canvassed by Dawson in preparation of his Infanticide Bill, summarised the position in an address to the same Society in 1941:

[I]t is still debatable whether the terms puerperal insanity and lactational insanity should be used and not exhaustion psychosis, whether the 1938 Act was needed or not, whether the Infanticide Act of 1922 plus the law of insanity were not sufficient to meet every kind of case that might come before the courts.^184^

Dr Norwood East concurred, concluding: ‘it was more desirable to leave these terms out entirely…it was ridiculous to talk about puerperal lactational insanity when it did not really describe the condition with which one was faced’.^185^

## Conclusion

On 7 March 1945, Viscount Dawson of Penn died. In testament to his status within the medical profession and his contribution to the nation, a memorial service was held at Westminster Abbey. As a giant of inter-war British medicine, various obituaries were dedicated to the physician. Yet his instrumental role in the passage of the Infanticide Act 1938 remained overlooked. As this article has shown, Dawson was central to this legislation. However, the reform of the 1922 Act was also built upon the back of a British medical profession that was deeply invested, and active in, reform of the law. A vocal British press, the campaigning efforts of professional women, and the contributions of other sympathetic parties, had enhanced awareness of infanticide as a crime that struck at the heart of formally respectable, industrious, or happy households by the early 1930s. Ordinarily good mothers were publicly shamed and torn asunder from family life by a criminal justice system that continued to be out of step with popular sympathies. This was especially true against the backdrop of the Hale case, in which Dawson gave expert defence testimony. Constructing infanticide as primarily a domestic tragedy was a strategy that he successfully deployed in his appeals within the Lords to justify both the timeliness, and necessity for, the reform of the 1922 Act.

That medicine should be deployed in service of the state, was a cherished belief the physician espoused publicly, and attempted to implement, through his 1937 Infanticide Bill. As a peer in the House of Lords and an expert witness, Dawson acted as a ‘medical statesman’ and representative of the wider profession. Situated against his life-long interests in population health, reproduction, and maternal welfare, infanticide reform was a logical conduit for his political energies during the late 1930s. Concerns over population decline and strategies to encourage women to embrace motherhood, frame Dawson’s involvement and outlook on the infanticide question with an urgency that appears not only distinctive to the physician’s own politics, but is also reflective of broader social and cultural anxieties about the future of the British nation in the inter-war years. His speeches in the Lords reified a belief that the insanity infanticidal women suffered from was largely transitory and symptomatic of an illness that could be recovered from. This accorded with the understanding attitudes adopted by most legal and medical practitioners, even if the medical profession at large remained divided ideologically on the existence of discrete reproductive insanities. While doctors like Summerskill emphasised the interplay between poverty and insanity as an exciting cause, other practitioners, like McIlroy and Dawson, held fast to the view that the physical strain of birth and childrearing triggered mental alienation and violence in some women. In Parliament, and before the British public, this discord was masked by an Infanticide Bill that consciously charted a middle course and conveyed an image of a united medical profession working in collaboration with the law to achieve a common good.

Although the Infanticide Act 1938 was viewed within the press and by many medical and legal professionals to be a just and measured reform, it was also not as ambitious or far-reaching as it could have been. Despite the impassioned arguments by some in the medical profession that infanticide reform was very much a *medical* issue and one for which doctors were ideally situated to contribute, Dawson was unable to secure the abolition of the verdict of ‘Guilty, but Insane’ or implement the recommendations of the Parliamentary Sub-Committee of the BMA. As with Summerskill and the editors of *The Lancet*, the BMA favoured greater involvement and increased powers for the psychiatric profession to promptly treat mentally disturbed women accused of infanticide. The hostility of government officials, the inability of reformist-minded doctors to translate desire into action, and the ongoing divide within the medical community over not only the need for infanticide legislation, but also how reproductive states impacted women’s health (and who was best-placed to decide), collectively thwarted a more overt medical model being implemented in the 1938 reforms, if not sooner. This was despite the eventually successful campaign being spearheaded by Dawson, a practitioner whose fame, connections, and commitment to a politics of ‘medical statesmanship’ preceded him. Against this backdrop and the earlier failures of more extreme proposals within the House of Commons, it is perhaps unsurprising that more expansive reforms never came to pass.

While some scholars have suggested that the medical footing of the Act was merely a tool deployed in service of the law, this position risks neglecting Dawson’s critical involvement in, and justifications for, reform in the interests of medicine. It also obscures the tangible energies members of the profession exercised in agitating on the infanticide question and in championing a more influential role for doctors within the criminal justice process. The physician’s sincere belief that medicine should serve the needs of the nation, and awareness that medical thinking (and ambitions) required adaptation to achieve change to the law, help explain why the eventual Act represented an amalgam of sympathetic medical, legal and lay thought on infanticide. As Dawson’s speeches and writings make clear, ‘solv[ing] the problems of life and health’ required both co-operation from politicians and the legal profession in changing the law, and the concession of medical ground.

## Footnotes

1 ‘Statement of Geoffrey Dyas’, 31 May 1936 in *Hale, Brenda: Murder and Attempted Suicide*, DPP Case File, (Kew: The National Archives (TNA), 1936), DPP 2/357, 17 & 19. NB. There is some discrepancy over Hale’s age in the source material. I have described her according to the legal record. This file is irregularly paginated; where possible, I have included page references.

2 ‘Copy of Statement of Charles Harold Medlock’, 31 May 1936 in *ibid.*, 3.

3 ‘Copy of Exhibit 2’ in *ibid.*

4 ‘Jury’s New Murder Verdict’, *The Daily Mail* (22 July 1936), 7.

5 As of 1936, his official title was “Bertrand Dawson, 1^st^ Viscount Dawson of Penn”.

6 The case was reported extensively within English newspapers, as well as in the *Edinburgh Evening News* and the *Belfast Telegraph.* It was also analysed in periodicals, e.g. ‘Medico-Legal: “Guilty but Insane”’, *British Medical Journal*, 2, 3943 (1 August 1936), 262; D. Seaborne Davies , ‘Child Killing in English Law: Part II’, Modern Law Review, 1, 4 (March 1938), 282–4 .

7 Infanticide Act, 1938.

8 Infanticide is not a distinct offence in Scotland. Instead, culpable homicide and concealment of pregnancy provide alternatives to murder. The Infanticide Act (Northern Ireland), 1939 was adopted in May of that year. Ireland passed its own Infanticide Act, modelled on the original 1938 legislation, in 1949.

9 Infanticide Act, 1922, c.18. s.1.

10 Historically, sentences for infanticide have included short terms of imprisonment or committal to a mental hospital, binding-over for a sum and probation.

11 Infanticide Act, 1938, c.26, s.1.

12

Michelle Oberman , ‘A Brief History of Infanticide and the Law’ in Margaret G. Spinelli (ed.), Infanticide: Psychosocial and Legal Perspectives on Mothers Who Kill (Washington & London: American Psychiatric Publishing, 2003), 9.

13 There is an extensive literature on this, for example: Susan S. M. Edwards , Women on Trial: A Study of the Female Suspect, Defendant and Offender in the Criminal Law and Justice System (Manchester: Manchester University Press, 1984), 4–13 , 79–100; Arlie Loughnan , ‘The ‘Strange’ Case of the Infanticide Doctrine’, Oxford Journal of Legal Studies, 32, 4 (2012), 685–711 . For recent feminist legal engagement with the gendered basis of the law and patriarchy, see particularly: Karen Brennan , ‘Murderous Mothers & Gentle Judges: Paternalism, Patriarchy, and Infanticide’, Yale Journal of Law and Feminism, vol. 30, 2018, pp. 139–95; Emma Milne and Karen Brennan , ‘The Infanticide Act 1938 as a Means to Provide Justice for Women Against the Hardships and Harms of Pregnancy and Motherhood?’, in Karen Brennan and Emma Milne (eds), 100 Years of the Infanticide Act: Legacy, Impact and Future Directions (London, Bloomsbury, 2023), 143–70.

14

Hilary Allen , Justice Unbalanced: Gender, Psychiatry, and Judicial Decisions (Milton Keynes: Open University Press, 1987), 28 .

15 See especially: James Mason , ‘The Myth of Madness: Murderous Mothers and Maternal Infanticide’, The Journal of Criminal Law, 85, 6 (2021), 441–54. Ronnie D. Mackay has argued for maintaining the Act based on an analysis of its operation under the CPS in the 1980s, see: R.D. Mackay, ‘The Consequences of Killing Very Young Children’, *Criminal Law Review* (January 1993), 21–30.

16

R. Kumar and Maureen Marks , ‘Infanticide and the Law in England and Wales’, in James Alexander Hamilton and Patricia Neel Harberger (eds), Postpartum Psychiatric Illness: A Picture Puzzle, (Philadelphia: University of Pennsylvania Press, 2016), 269 .

17

Karen Brennan and Emma Milne , ‘Criminalising Neonaticide: Reflections on Law and Practice in England and Wales’, in Emma Milne , et al. (eds), Women and the Criminal Justice System: Failing Victims and Offenders? (Cham: Palgrave Macmillan, 2018), 106 .

18 Rachel Dixon and Tony Ward, ‘Manslaughter, Concealment of Birth and Infanticide, 1900-37’, in Brennan and Milne (eds), *op. cit.* (note 13), 49–65; Donna Cooper Graves , ‘“…in a frenzy while raving mad”: Physicians and Parliamentarians Define Infanticide in Victorian England’, in Brigitte H. Bechtold & Donna Cooper Graves (eds), Killing Infants: Studies on the Worldwide Practice of Infanticide (Lewiston: Edwin Mellen Press, 2006), 111–35; Daniel J.R. Grey , ‘Women’s Policy Networks and the Infanticide Act 1922’, Twentieth Century British History, 21, 4 (2010), 441–6321465999 10.1093/tcbh/hwq024; Nigel Walker , Crime and Insanity in England: Vol.1 The Historical Perspective (Edinburgh: Edinburgh University Press, 1968), 125–37.

19 For example: Donald Nicholson , ‘What the Law Giveth, It Also Taketh Away: Female-Specific Defences to Criminal Liability’, in Lois Bibbings and Donald Nicolson (eds), Feminist Perspectives on Criminal Law (London: Cavendish, 2000), 162 .

20

Tony Ward , ‘Legislating for Human Nature: Legal Responses to Infanticide, 1860-1938’, in Mark Jackson (ed.), Infanticide: Historical Perspectives on Child Murder and Concealment, 1550-2000 (Aldershot & Burlington: Ashgate, 2002), 268 .

21

Tony Ward , ‘The Sad Subject of Infanticide: Law, Medicine and Child Murder, 1860-1938’, Social & Legal Studies, 8, 2 (1999), 174–6.

22
*Ibid.*, 174.

23 On this latter point, see: Hilary Marland , Dangerous Motherhood: Insanity and Childbirth in Victorian Britain (Basingstoke & New York: Palgrave Macmillan 2004), 200–9.

24

Kirsten Johnson Kramar & William D. Watson , ‘The Insanities of Reproduction: Medico-Legal Knowledge and the Development of Infanticide Law’, Social & Legal Studies 15, 2 (2006), 237–55.

25
*Ibid.*, 251.

26
*Ibid.*

27

Karen Brennan , ‘Beyond the Medical Model: A Rationale for Infanticide Legislation’, Northern Ireland Legal Quarterly, 58, 4 (2007), 533 .

28

Rachel Dixon , Infanticide: Expert Evidence and Testimony in Child Murder Cases, 1688-1955 (Milton: Taylor & Francis, 2021), 145–9.

29 There is an extensive literature on this topic, for example: George K. Behlmer , ‘Deadly Motherhood: Infanticide and Medical Opinion in Mid-Victorian England’, Journal of the History of Medicine and Allied Sciences, 34, 4 (1979), 403–27389999 10.1093/jhmas/xxxiv.4.403; Ruth Ellen Homrighaus , ‘Wolves in Women’s Clothing: Baby-Farming and the *British Medical Journal*, 1860-1872’, Journal of Family History, 26, 3 (2001), 350–7219320077 10.1177/036319900102600303; Mark Jackson , ‘Suspicious Infant Deaths: The Statue of 1624 and Medical Evidence at Coroners’ Inquests’ in Michael Clark and Catherine Crawford (eds), Legal Medicine in History (Cambridge, Cambridge University Press, 1994), 64–86 .

30

Joel Peter Eigen , Witnessing Insanity: Madness & Mad-Doctors in the English Court (New Haven & London: Yale University Press, 1995), 142.

31

Anne-Marie Kilday , A History of Infanticide in Britain, c. 1600 to the Present, (Basingstoke & New York: Palgrave, 2013), 170 .

32 Marland, *op. cit.* (note 23), 182–3.

33
*Ibid.*, 183.

34
*Ibid.*, 178.

35 A particularly good example of psychiatric evidence being used in infanticide prosecutions can be found in McKay, *op. cit.* (note 15), 21–30.

36 Law Commission, *Murder, Manslaughter and Infanticide* (London, TSO, 2006, Law Com No. 304), 156 – 71. The 2006 Law Commission’s consultation on infanticide followed the 2005 Labour government announcement of a review of the law of murder in England and Wales. The consultation on infanticide canvassed views from law, academia, medicine, the police, ‘interested organisations’ and the public. Individual psychiatrists, such as Professor Ian Brockington and Dr Maureen Marks, were consulted, as were representative medical bodies, such as the Royal College of Psychiatrists.

37
*Ibid.*, 161.

38 In addition to surveying the holdings of the Wellcome Library (including – but not limited to – the surviving records held within the Dawson collection, see: *Bertrand Edward Dawson, Viscount Dawson of Penn*, (London: Wellcome Library, 1886-1945), PP/BED) and the RCP (including – but not limited to – the *Minutes of the Council of the RCP*, (London: RCP, 1926 – 1942), MS 4681 – 3) the author also searched the holdings of the UK Parliamentary Archives for material related to the introduction of Dawson’s Infanticide Bill and the parliamentary response it elicited. Unfortunately, this survey yielded no additional relevant material and underscored that the best insight into the reception to the Bill by Parliament remains that offered in Hansard. Dawson’s biography gives little insight as to why he took up this campaign. See: Francis Watson , Dawson of Penn (London: Chatto & Windus, 1950), 243–5. For a recent scholarly treatment of Dawson’s role in infanticide reform, see: Karen Brennan and Emma Milne, ‘Introduction’ in Brennan & Milne (eds), *op. cit.* (note 13), 13–14.

39 Some key works to have analysed Dawson’s life and career include: G.C. Cook , ‘The Practice of Euthanasia at the Highest Level of Society: The Lords Dawson (1864-1945) and Horder (1871-1955)’, Journal of Medical Biography, 14, 2 (2006) 90–216607408 10.1258/j.jmb.2006.05-36; Charles Webster , ‘The Metamorphosis of Dawson of Penn’, in Dorothy Porter and Roy Porter (eds), Doctors, Politics and Society: Historical Essays (Leiden: Brill, 1993), 212–26; Claire L. Jones , The Business of Birth Control: Contraception and Commerce in Britain Before the Sexual Revolution (Manchester: Manchester University Press, 2020), 172–204 .

40 Jones, *ibid.*,197.

41

Jane Carey , ‘The Racial Imperatives of Sex: Birth Control and Eugenics in Britain, the United States and Australia in the Interwar Years’, Women’s History Review, 21, 5 (2012), 746 .

42

Marie Carmichael Stopes , Contraception (Birth Control): Its Theory, History and Practice (London: John Bales, Sons & Danielsson, Ltd, 1927), 27–35 especially.

43 For an excellent overview of illegitimacy in Britain, see: Jane Robinson , In the Family Way: Illegitimacy between the Great War and the Swinging Sixties (London: Penguin Books, 2016).

44 Infant Life Preservation Act (1929), s. 1.

45

Stephen Brooke , ‘“A New World for Women”? Abortion Law Reform in Britain During the 1930s’, The American Historical Review, 106, 2 (2001), 449–5018186164.

46 For example: A. Louise McIlroy , ‘Some Factors in the Control of the Birth-Rate’, Transactions of the Medico-Legal Society, 15, 1 (1921-1922), 137–53; Lord Riddell , ‘Ancient Laws and Modern Women: Presidential Address’, Transactions of the Medico-Legal Society, 24 (1929), 1–19 .

47

Jay Murray Winter , ‘Infant Mortality, Maternal Mortality and Public Health in Britain in the 1930s’, Journal of European Economic History, 8, 2 (1979), 439–6211614742.

48

Jane Lewis , ‘The Problem of Lone-Mother Families in Twentieth-Century Britain’, The Journal of Social Welfare & Family Law, 20, 3 (1998), 251–83.

49 Kilday, *op. cit.* (note 31), 151–82.

50 Walker, *op. cit.* (note 18), 127–9.

51 Ward, *op. cit.* (note 21) 165.

52 Behlmer, *op. cit.* (note 29), 412–3.

53 For a useful historical criticism of judicial sentencing in infanticide cases, see: Davies, *op. cit.* (note 6), 284–5.

54 Ward, *op. cit.* (note 20), 257–61.

55 Grey, *op. cit.* (note 18), 441–63; Ann Logan , Feminism and Criminal Justice: A Historical Perspective (London: Palgrave Macmillan, 2008), 130–8. NB. Although sentenced to death, Roberts was later reprieved.

56 HC Debs (13 February 1922), series 5, vol.150, col.616.

57 Ward, *op. cit.* (note 21), 170.

58 Infanticide Act 1922, s 1.

59 ‘Most Wise New Act’, *Daily Mirror*, (31 October 1922), 2.

60 ‘The Infanticide Act’, *Gloucester Journal*, (17 January 1925), 9.

61
*R. v Chick* (Florence Irene) (1927) 19 Cr. App. R., 57–8.

62
*Ibid.*, 58.

63 ‘An example of the manner…’, Untitled Memorandum, (n.d.) in *Effects of the Act: Tables of Crimes and Convictions, 1921 – 1933* (Kew: TNA, 1925-33), HO 329/119, 3. This file is irregularly paginated; where possible, I have included page references.

64 ‘Medico-Legal Notes: Rex *v.* Mary O’Donoghue’, *Journal of Mental Science*, 74, 304 (1928), 100.

65 R. *v* O’Donoghue (Mary), (1928) 20 Cr. App. R., 136.

66 ‘Medico-Legal Notes’, *op. cit.* (note 64), 100.

67

A. Louise McIlroy , ‘The Influence of Parturition Upon Insanity and Crime’, Transactions of the Medico-Legal Society, 22 (1927-1928), 53 10.1136/bmj.1.3503.303PMC245525020773708.

68 McIlroy, *op. cit.* (note 6), 137–53; Louise McIlroy , The Toxaemias of Pregnancy (London: Edward Arnold & Co, 1936).

69 McIlroy, *op. cit.* (note 67), 57–61.

70
*Ibid.*, 55, 58–9.

71
*Ibid.*, 60.

72
*Ibid.*

73
*Ibid.*, 59.

74 Humphreys quoted in: *Ibid.*, 63.

75 Stopes & Worth in: *Ibid.*, 71–2.

76
*Ibid.*

77
*Ibid.*, 57.

78
*Ibid.*

79
*Ibid.*, 60.

80

J.M. Munro Kerr et al., Combined Textbook of Obstetrics and Gynaecology: For Students and Medical Practitioners, 4th edn (Edinburgh, E. & S. Livingstone, 1928), 255–8 especially. This edition was revised by eminent Scottish practitioner of midwifery and obstetric surgery, John Martin Munro Kerr – and featured the contributions of eight other experts in inter-war obstetrics and gynaecology.

81 McIlroy quoted in: McIlroy, *op. cit.* (note 67), 63–5.

82 Bond quoted in: *Ibid.*, 64.

83
*Ibid.*, 64–5.

84

W. Norwood East , Introduction to Forensic Psychiatry (London: J & A Churchill, 1927).

85 McIlroy quoted in: McIlroy, *op. cit.* (note 67), *72.*

86
*Ibid.*, 73.

87

A.L. McIlroy , ‘The Influence of Parturition Upon Insanity and Crime’, British Medical Journal, 1, 3503 (1928), 303–420773708 10.1136/bmj.1.3503.303PMC2455250; A. Louise McIlroy , ‘The Influence of Parturition Upon Insanity and Crime: An Address to the Medico-Legal Society, Feb. 23rd, 1928’, The Lancet, 211, 5452 (1928), 379–81.

88

K. Crosby , ‘Abolishing Juries of Matrons’, Oxford Journal of Legal Studies, 39, 2 (2018), 259–84.

89 Sentence of Death (Expectant Mothers) Act, 1931, s. 1.

90 ‘Justice for Law’s Victim’, *North East Daily Gazette* (2 September 1931); ‘Jailed Mother of 14 Children’, *Daily Herald* (2 September 1931) in *Conservative Party Press Cuttings – Ellen Wilkinson, Labour Politician*, (University of Warwick: Modern Records Centre (MRC), 1931), MSS.209/21/1. NB. Original not paginated.

91 ‘Editorial: Sir Herbert Samuel…’, *Daily Express* (11 September 1931) in *Criminal: Woman Sentenced to 3 Months’ Imprisonment for Attempted Suicide* (Kew: TNA, 1931-32), HO 144/16353. NB. Original not paginated.

92 ‘Mother Freed after 10 Days’, *Daily Mail* (11 September 1931) in *ibid.*

93

E.W. Anderson , ‘A Study of the Sexual Life in Psychoses Associated with Childbirth’, Journal of Mental Science, 79, 324 (1933), 137 .

94

A. Leyland Robinson , ‘The Effect of Reproduction Upon Insanity’, British Journal of Gynaecology, 40, 1 (1933), 39–66 .

95
*Ibid.*, 41.

96
*Ibid.*, 46–7.

97
*Ibid.*, 40.

98
*Ibid.*, 49.

99
*Ibid.*

100 ‘Infanticide and Unemployment’, *The Manchester Guardian* (2 September 1935), 10.

101
*Ibid.*

102 ‘Unusual Verdict in Murder Trial’, *Daily News* (22 July 1926), 7.

103 ‘Tewin Farmer’s Wife on Murder Charge’, *Hertfordshire Express* (11 July 1936), 2.

104
*Ibid.*; ‘Letter from Alexander Maxwell to Unknown Recipient (likely Schuster)’, 28 July 1936 in *Criminal: Infanticide Acts*, *1922 and 1938*, (Kew: TNA, 1922-43), HO45/19230. NB. This file is irregularly paginated; where possible, I have included page references.

105 Henry Bashford, ‘Dawson of Penn’, *The Spectator* (16 March 1945), 241.

106 ‘Obituary’, *The Manchester Guardian* (8 March 1945), 3.

107 ‘Obituary’, *British Medical Journal*, 4393 (17 March 1945), 389.

108
*Ibid.*, 390–1.

109 ‘Obituaries: Lord Dawson of Penn’, *Nature*, 155, 3933 (1945), 325.

110 ‘Training in Crime Detection’, *The Times* (25 July 1936), 4; ‘The Medico-Legal Society’s Annual Dinner’, *Medico-Legal and Criminological Review*, 7, 1 (January 1939), 63–71.

111 Lord Dawson of Penn, ‘Medicine & Statesmanship’, *The Atlantic Monthly*, CXLVII (January-June 1931), 210.

112 Lord Dawson of Penn, *Love-Marriage-Birth Control* (London: Nisbet & Co., 1922). Dawson’s speech was republished by the Birth Control Review in New York in 1922.

113
*Ibid.*, 25–6.

114 Quoted in: *Ibid.*, 7 & 26. For this sentiment repeated in the 1930s, see: “No Family Should Be Fewer Than 3’, *Daily Mail* (24 May 1939), 5.

115 For a good discussion of this theme and interwar eugenics, see: Richard Overy , The Morbid Age: Britain and the Crisis of Civilization (London: Penguin Books, 2010), 93–135 .

116 Lord Dawson of Penn, ‘Address to York Medical Society on Medical Science and Social Progress’, *British Medical Journal*, 2, 3904, (1935), 830–1.

117
*Ibid.*, 832. For Dawson’s involvement in the latter campaign, see: ‘Minutes & Papers of the Voluntary Sterilisation Committee’ in *Voluntary Sterilisation Committee* (London, RCP, 1934-1939), MS.5074.

118 For example: Helen Simpson, “They should have more babies’ says Lord Dawson’, *Daily Mail* (25 May 1939), 10.

119 ‘A Brace of Babies’, *Punch* (7 July 1937), 17.

120 ‘Medico-Legal’, *op. cit.* (note 6), 262.

121 Dawson quoted in: HL Debs (22 March 1938), series 5, vol.108, col.294.

122 ‘Farmer’s Wife Not Guilty of Murder’, *Hertforshire Express* (25 July 1936), 11.

123 ‘Murder Trial Ruling’, *Liverpool Echo* (21 July 1936), 7.

124 Humphreys quoted in: ‘Infanticide: Judge and the Law’, *The Times* (22 July 1936), 13.

125 For example: ‘Unusual Verdict in Murder Trial’, *op. cit.* (note 102), 7.

126 ‘Reprieve Comes as Mother Says Good-Bye: Two Fathers in Prison Cell Dramas’, *Daily Herald* (22 July 1936), 9.

127 ‘Mr Rhys Davies: Case of Mrs Brenda Hale’, 28 July 1936 in *Criminal: Infanticide Acts*, *op. cit.* (note 104).

128
*Criminal: Infanticide Acts*, *op. cit.* (note 104); *Infanticide: Proposed Amendment of Infanticide Act*, *1922* (Kew: TNA, 1936-38), LCO 2/1329. NB. This file is irregularly paginated, where possible I have included page references.

129
*Minute Book*, 1933-46 in *Collections of the National Association for the Abolition of Capital Punishment* (University of Warwick: MRC, 1923-49), MSS.16B/ADP/1/5.

130 ‘Extract from 12 & 13 Geo.5 cap.18’, 19 February 1936 in *Criminal: Infanticide Acts*, *op. cit.* (note 104).

131 HC Debs (24 November 1936), series 5, vol. 318, cols. 234-236.

132 ‘Letter from Alexander Maxwell to Claud Schuster’, 25 November 1936 in *Infanticide: Proposed Amendment of Infanticide Act*, *op. cit.* (note 128).

133 ‘Letter from Travers Humphrey to the Lord Chancellor’, 22 July 1936 in *ibid.*

134 ‘Letter from Claud Schuster to Travers Humphrey’, 23 July 1936 in *ibid.*,1.

135 ‘Letter from Claud Schuster to Alexander Maxwell’, 30 July 1936 in *Criminal: Infanticide Acts*, *op. cit.* (note 104), 2.

136
*Ibid.*, 1.

137 ‘Letter from Claud Schuster to Travers Humphrey’ in *Infanticide: Proposed Amendment of Infanticide Act*, *op. cit.* (note 128), 1–2.

138 ‘Letter from Alexander Maxwell to Claud Schuster’, 25 November 1936 in *ibid.*

139 ‘Letter from Travers Humphrey to the Lord Chancellor’, in *ibid.*, 2.

140
*Ibid.*

141 ‘Memorandum by Alexander Maxwell’, 28 July 1936 in *Criminal: Infanticide Acts*, *op. cit.* (note 104), 1.

142 ‘Safer Motherhood’, *The Times* (26 October 1936), 11.

143 ‘Minute by Edward Hale Tindal Atkinson, DPP’, 22 September 1936 in *Infanticide: Proposed Amendment of Infanticide Act*, *op. cit.* (note 128).

144 ‘Seven Years P.R.C.P’, *The Lancet*, 231, 5981 (16 April 1938), 899.

145 Dawson quoted in: HL Debs, *op. cit.* (note 121), col. 308; HL Debs (7 April 1938), series 5, vol. 108, col. 600.

146

J.C.M. Matheson , ‘Infanticide’, Medico-Legal and Criminological Review, 9, 3 (July 1941), 142 . Matheson’s potential involvement in the ‘discussions leading to Dawson’s Bill’ has also been noted in Ward, *op. cit.* (note 21), 177, note 5. However, Ward does not speculate further on the significance of this point. Nevertheless, he does suggest that Matheson would likely have been sceptical of the biomedical justifications for the Bill.

147 ‘Medical Report of Dr J.C.M. Matheson, HMP Holloway’, 13 July 1936 in *Hale, Brenda*, *op. cit.* (note 1).

148 Stephen Watson, ‘Malingerers, the ‘Weakminded’ Criminal and the ‘Moral Imbecile’: How the English Prison Medical Officer Became an Expert in Mental Deficiency, 1880-1930," in Clark & Crawford (eds), *op. cit.* (note 29), 223–41.

149 ‘Letter from A.E.W. Petrie to Dr Foulerton’, 28 March 1938 in *Criminal: Infanticide Acts*, *op. cit.* (note 104).

150 For example: ‘Mercy for Mothers’, *Daily Mail* (23 March 1938), 9; ‘Saving the Mothers’, *Nottingham Journal* (23 March 1938), 7.

151 Dawson quoted in: HL Debs, *op. cit.* (note 121), col.309.

152
*Ibid.*, col.291.

153
*Ibid.*, cols. 294–6.

154
*Ibid.*, col.296.

155
*Ibid.*, col.294.

156
*Ibid.*, col.295.

157 Dawson quoted in HL Debs, *op. cit.* (note 145), cols. 599–600.

158
*Ibid.*, col. 600.

159 ‘Minute by Edward Hale Tindal Atkinson’, 10 January 1938 in *Criminal: Infanticide Acts, op. cit.* (note 104), 1.

160 Dawson quoted in: HL Debs, *op. cit.* (note 145), cols. 601–2.

161 ‘Letter from Alexander Maxwell to Sir Donald Somervell, Attorney-General’, 9 March 1938 in *Criminal: Infanticide Acts*, *op. cit.* (note 104).

162 ‘Letter from Claud Schuster to Alexander Maxwell’, 8 February 1938 in *Infanticide: Proposed Amendment of Infanticide Act, op. cit.* (note 128).

163 ‘Lords Support Infanticide Bill’, *Daily Telegraph* (23 March 1938) in *Criminal: Infanticide Acts*, *op. cit.* (note 104).

164 ‘A Law of Mercy’, *Daily Express* (23 March 1938) in *Criminal: Infanticide Acts*, *op. cit.* (note 104).

165 Gilbert Frankau, ‘I Would Punish Women Equally with Men’, *Daily Mail* (24 March 1938), 10.

166
*Ibid.*

167 ‘Infanticide’, *Law Journal*, LXXXV, 3756 (8 January 1938), 23.

168

H.A. Palmer , ‘Infanticide’, Justice of the Peace & Local Government Review, 102, 6 (5 February 1938), 85 .

169 ‘Infanticide’, *Medico-Legal and Criminological Review*, 6, 2 (1938), 174.

170
*Ibid.*

171 Palmer, *op. cit.* (note 168), 85.

172 ‘Infanticide’, *The Lancet*, 1, 5959 (22 January 1938), 211.

173
*Ibid.*

174
*Ibid.*, 212.

175 ‘The Medico-Political Committee of the B.M.A’, *British Medical Journal*, 1, 4038 (1938), 336.

176 ‘File Note by Chief Magistrate’, 27 July 1938 in *Criminal: Infanticide Act, op. cit.* (note 104).

177 A.B. Austin, ‘Surgeon Not Guilty’, *Daily Herald* (20 July 1938), 1. Bourne was tried for unlawfully using an instrument to procure abortion on a 14-year-old girl who had become pregnant following a rape.

178 Dawson quoted: in HL Debs (21 June 1939), series 5, vol.113, col.619.

179
*Ibid.*, cols. 622–5.

180
*Ibid.*, col. 626.

181 Extracted from: ‘Table B’, *Criminal Statistics England and Wales 1950* (London: HMSO, 1951), 6.

182 ‘Crisis Fears Made Mother Kill Baby’, *Sunderland Daily Echo and Shipping Gazette* (17 November 1938), 13.

183 ‘Liverpool Murder Charge Fails’, *Evening Express* (19 October 1939), 4.

184 Matheson, *op. cit.* (note 146), 143.

185 East quoted in: *Ibid.*, 148.

